# Geographic Variations and Time Trends in Cancer Treatments in Taiwan

**DOI:** 10.1186/s12889-017-4615-y

**Published:** 2017-08-02

**Authors:** Jason C. Hsu, Sheng-Mao Chang, Christine Y. Lu

**Affiliations:** 1 School of Pharmacy and Institute of Clinical Pharmacy and Pharmaceutical Sciences, College of Medicine, National Cheng Kung University, No.1, Daxue Rd., East Dist., Tainan, 70101 Taiwan; 2 Department of Statistics, College of Management, National Cheng Kung University, Tainan, Taiwan; 3 Department of Population Medicine, Harvard Medical School and Harvard Pilgrim Health Care Institute, Boston, MA USA

**Keywords:** Cancer, Targeted Therapy, Geographic regions, Taiwan

## Abstract

**Background:**

Targeted therapies have become important treatment options for cancer care in many countries. This study aimed to examine recent trends in utilization of antineoplastic drugs, particularly the use of targeted therapies for treatment of cancer, by geographic region in Taiwan (northern, midwestern, southern, and eastern regions and the outer islands).

**Methods:**

This was a retrospective observational study of antineoplastic agents using 2009-2012 quarterly claims data from Taiwan’s National Health Insurance Research Database. Yearly market shares by prescription volume and costs for targeted therapies among total antineoplastic agents by region were estimated. We used multivariate regression model and ANOVA to examine variations in utilization of targeted therapies between geographic regions and used ARIMA models to estimate longitudinal trends.

**Results:**

Population-adjusted use and costs of antineoplastic drugs (including targeted therapies) were highest in the southern region of Taiwan and lowest in the outer islands. We found a 4-fold difference in use of antineoplastic drugs and a 49-fold difference in use of targeted therapies between regions if the outer islands were included. There were minimal differences in use of antineoplastic drugs between other regions with about a 2-fold difference in use of targeted therapies. Without considering the outer islands, the market share by prescription volume and costs of targeted therapies increased almost 2-fold (1.84-1.90) and 1.5-fold (1.26-1.61) respectively between 2009 and 2012. Furthermore, region was not significantly associated with use of antineoplastic agents or use of targeted therapies after adjusting for confounders. Region was associated with costs of antineoplastic agents but it was not associated with costs of targeted therapies after confounding adjustments.

**Conclusions:**

Use of antineoplastic drugs overall and use of targeted therapies for treatment of cancer varied somewhat between regions in Taiwan; use was notably low in the outer islands. Strategies might be needed to ensure access to cancer care in each region as economic burden of cancer care increase due to growing use of targeted therapies.

## Background

Knowledge of etiology and epidemiology of cancer has great importance for control of cancer, which is a major public health issue across the world due to its high mortality and increasing prevalence [1, 2]. Over the past few decades, a considerable number of studies have examined factors associated with the occurrence, treatment and outcomes of specific cancer type in many countries [3–5]. Demographic characteristics (including hereditary genetic mutations, age, gender, ethnicity, education, and geographic location), environmental hazards (such as exposure to radiation, smoking, and pollutions), patient health status and behavior (e.g., diet, obesity, physical activity, history of disease, comorbidity and drug use), and health care providers’ characteristics (e.g., physicians’ knowledge and preferences, prescribing patterns, clinical guidelines and economical considerations) may contribute to variations in cancer incidence, diagnosis, treatment and outcomes between regions [6, 7]. Better understanding of factors influencing cancer diagnosis and treatments can allow us to improve quality of cancer care and outcomes and facilitate health policy planning.

In the last few years, several studies have assessed the association between geographic variation and incidence of cancer [8–12]. For instance, a US study [12] found that incidence of colorectal cancer was highest in the Middle Atlantic division, with the lowest rate observed in the Mountain division. Researchers have investigated cancer screening or diagnosis in various geographic locations, and found geographic variations in the uptake of screening and diagnosis of several cancers including cervical, lung and liver cancers [13–15]. Such geographical differences have increased during the past decade in many countries, including Taiwan [13–17]. Cancer treatment and cancer care also vary between geographic locations [10, 18–21]. For example, Reames et al. [21] reported wide geographic variations in utilization of laparoscopic colectomy among US Medicare patients with colon cancer (from 0% to 66.8% across 306 hospital referral regions). Furthermore, the association between geographic variation and clinical outcomes (such as survival and mortality) of cancer treatment has been investigated extensively [8, 9, 22, 23]. Andia et al. [23] indicated that gallbladder cancer mortality rate was higher in the inland and southern regions of Chile, and compared to the north-coastal region, the northern-inland region had a 10-fold higher risk and the southern-inland region had a 26-fold higher risk.

There has been an increasing availability and use of targeted therapies for treatment of cancer due to their high treatment response rates [24, 25] but less toxic characteristics compared with traditional chemotherapies [26]. For several cancers, targeted therapies are becoming the main treatments; examples include erlotinib for lung cancer [27–29] and trastuzumab for breast cancer [30, 31]. With the growing trends in use of targeted therapies, the burden of pharmaceutical expenditure due to their high costs and their accessibility, have lately become one of the most serious concerns in all countries with universal health insurance systems, including Taiwan’s national health insurance system. Taiwan’s national health insurance system is a compulsory social insurance system in which the coverage rate of its 23 million residents is as high as 99% currently [32].

Previous research has not addressed whether or not the use of targeted therapies for treatment of cancer differs from region to region in Taiwan [33, 34]. To provide insights about the accessibility and equity issues related to high-cost targeted therapies, we examined use and costs of antineoplastic agents by geographic region (northern, midwestern, southern, and eastern regions, and the outer islands) in Taiwan, particularly focusing on cancer targeted therapies.

## Method

### Data sources

This study used nationwide claims data from the National Health Insurance Research Database (NHIRD), which compiles data of over 99% of population (around 23 million residents) in Taiwan. The database contains information from a mandatory-enrollment and single-payer healthcare system created in 1995, and it covers a wide range of prescription medicines, and inpatient and outpatient medical services [35]. We obtained 2009-2012 quarterly claims data from NHIRD regarding treatment of malignancies, including details of prescriptions and national health insurance expenditure for antineoplastic agents. The cancer related prescriptions were identified using International Classification of Diseases, 9^th^ edition (ICD-9) diagnosis codes (codes: 140-239). We converted costs in Taiwan dollars to US dollars by 30:1. Our previous studies also used this data source [36, 37]. In addition, yearly population data by age and region, yearly geographical area by region, yearly number of physician by region were obtained from the Department of Statistics, Taiwan Ministry of Interior [38]. We obtained population statistics and cancer incidence from Taiwan Cancer Registry Annual Report [39].

### Drugs included

We used World Health Organization’s Anatomical Therapeutic Chemical (ATC) classification system to identify antineoplastic agents (with code “L01”). We grouped antineoplastic agents into 6 classes based on the ATC system: [1] targeted therapies (including monoclonal antibodies, protein kinase inhibitors and other targeted therapies). Among targeted therapies: monoclonal antibodies (rituximab, trastuzumab, and cetuximab), protein kinase inhibitors (imatinib, gefitinib, erlotinib, sunitinib, sorafenib, dasatinib, nilotinib, temsirolimus, everolimus, and pazopanib), and bortezomib have been used for the treatment of cancer in Taiwan; [2] alkylating agents (including nitrogen mustard analogues, alkyl sulfonates, nitrosoureas and other alkylating agents); [3] antimetabolites (including folic acid analogues, purine analogues and pyrimidine analogues); [4] plant alkaloids and other natural products (including vinca alkaloids and analogues, podophyllotoxin derivatives and taxanes); [5] cytotoxic antibiotics and related substances (including actinomycines, anthracyclines and related substances and other cytotoxic antibiotics); and [6] other agents (including platinum compounds, sensitizers used in photodynamic/radiation therapy and others).

### Outcome measures

We calculated quarterly and yearly number of prescriptions and costs from 2009 to 2012 to examine the use and costs of each class of antineoplastic agents. We used administrative divisions of Taiwan to group utilization and costs according to the 5 regions: northern, midwestern, southern, and eastern regions and the outer islands (Appendix 1). To assess regional differences, we estimated population-adjusted number of targeted therapies prescription and costs (per 100,000 people) for each region. For each region, population-adjusted prescription volume was calculated by using number of prescriptions divided by number of population in that region, and population-adjusted costs was calculated by using total costs divided by number of population in the region. Because cancer incidence by year and across regions was overall stable (Appendix 2), we presented population-adjusted results by region.

We also calculated the yearly market shares by prescription volume and by costs for targeted therapies among total antineoplastic agents. Market share by prescription volume was estimated by: number of prescriptions for targeted therapies divided by total number of prescriptions for all antineoplastic agents. The market share by costs was estimated by: costs of targeted therapies divided by total costs of all antineoplastic agents.

### Statistical Analysis

We used univariate and multivariate regression models and analysis of variance (ANOVA) to examine the association between ‘region’ and population-adjusted use and costs of antineoplastic agents. In separate models, we assessed the association between ‘region’ and population-adjusted use and costs of targeted therapies. We only focused on four regions - northern, midwestern, southern and eastern regions; we excluded the outer islands in these models because its use of antineoplastic agents was much lower than that in other regions. Our models controlled for the following covariates: average age of cancer patients in that region, percentage of male cancer patients in that region, population density (number of population divided by geographic area), physician density (number of physician divided by number of population in the region * 100,000 people), and cancer incidence per 100,000 population. We also used a time series design with the Autoregressive integrated moving average (ARIMA) model [40, 41] to estimate longitudinal trends in targeted therapy use and costs. All analyses were carried out with SAS software, Version 9.3 (SAS Institute, Cary, NC).

## Results

### Regional variations

Between 2009 and 2012, we observed steady increases in use of all antineoplastic drugs in all regions (ranged from 1.33 to 1.56-fold increase in population-adjusted prescription volume). Use of cancer targeted therapies also more than doubled in all regions except the outer islands (ranged from 2.47 to 2.54-fold increase in population-adjusted prescription volume across regions without considering the outer islands). Table 1 presents use and costs by region across years. In 2012, there was a 4.20-fold difference in population-adjusted use of all antineoplastic drugs between the southern region (highest) and the outer islands (lowest) but a 49.11-fold difference in population-adjusted use of targeted therapies between the southern region (highest) and the outer islands (lowest). Without considering the outer islands, there was a 1.25-fold difference in population-adjusted use of antineoplastic drugs between the southern region and the eastern region and a 1.89-fold difference in population-adjusted use of targeted therapies between the southern region and the eastern region in 2012.

**Table 1:** Yearly population-adjusted prescription volume and costs of antineoplastic agents by region (2009-2012)

Region	Year	Population	Prescription volume and cost of targeted therapies and all antineoplastic agents							
			Number of prescriptions of TT	Number of prescriptions of all antineoplastic agents	Population adjusted prescription volume of TT per 100,000 people	Population adjusted prescription volume of all antineoplastic agents per 100,000 people	Cost of TT (US$)	Cost of all antineoplastic agents (US$)	Population adjusted Cost of TT per 100,000 people	Population adjusted Cost of all antineoplastic agents per 100,000 people
Northern	2009	10,794,022	41,563	1,200,447	385.06	11,121.41	44,727,732.83	245,327,405.57	414,375	2,272,808
	2010	10,852,146	53,085	1,352,126	489.17	12,459.53	64,066,867.43	298,340,256.13	590,361	2,749,136
	2011	10,919,415	74,409	1,539,869	681.44	14,102.12	80,098,867.07	335,228,189.63	733,545	3,070,020
	2012	10,991,480	107,447	1,681,041	977.55	15,294.04	108,328,729.00	369,543,104.87	985,570	3,362,087
Midwestern	2009	5,201,847	16,182	517,142	311.08	9,941.51	17,550,734.80	105,008,551.23	337,394	2,018,678
	2010	5,199,849	22,116	587,463	425.32	11,297.69	25,919,867.20	130,844,276.37	498,473	2,516,309
	2011	5,203,796	28,424	641,058	546.22	12,319.05	31,768,038.03	148,131,812.10	610,478	2,846,611
	2012	5,215,948	41,136	691,949	788.66	13,266.03	45,052,172.70	168,569,414.17	863,739	3,231,808
Southern	2009	6,446,720	26,808	735,122	415.84	11,403.04	27,431,583.03	143,013,766.23	425,512	2,218,396
	2010	6,433,342	33,836	834,331	525.95	12,968.86	37,988,591.17	178,074,087.10	590,495	2,767,987
	2011	6,422,584	45,262	904,722	704.73	14,086.57	48,387,511.27	201,906,589.30	753,396	3,143,697
	2012	6,422,531	65,905	983,272	1,026.15	15,309.73	66,284,538.63	223,610,506.80	1,032,063	3,481,657
Eastern	2009	573,461	1,255	52,193	218.85	9,101.40	1,872,531.70	9,729,753.87	326,532	1,696,672
	2010	569,478	1,736	60,197	304.84	10,570.56	2,248,463.80	12,178,410.27	394,829	2,138,522
	2011	565,128	2,415	66,179	427.34	11,710.44	2,951,519.27	14,101,363.17	522,274	2,495,251
	2012	561,442	3,047	68,875	542.71	12,267.52	3,493,181.90	14,396,745.00	622,180	2,564,244
Outer Islands	2009	103,722	1	2,431	0.96	2,343.77	53.30	170,837.17	51	164,707
	2010	107,308	0	3,261	0.00	3,038.92	0.00	316,458.37	0	294,907
	2011	113,989	1	3,654	0.88	3,205.57	649.13	387,676.10	569	340,100
	2012	124,421	26	4,539	20.90	3,648.10	17,180.40	481,334.53	13,808	386,860

*TT* targeted therapies; Population adjusted prescription volume = Number of prescriptions / Population, per 100,000 people; Population adjusted costs = Cost / Population, per 100,000 people

Similarly, population-adjusted costs of all antineoplastic drugs increased from 2009 to 2012 in all regions (ranged from 1.48 to 2.35-fold increase in population-adjusted costs). Costs of cancer targeted therapies also more than doubled in most regions (ranged from 1.91 to 2.56-fold increase in population adjusted costs across regions without considering the outer islands). In 2012, there was a 9.00-fold difference in population-adjusted costs of antineoplastic drugs between the southern region (highest) and the outer islands (lowest) and a 74.74-fold difference in population-adjusted costs of targeted therapies between the southern region (highest) and the outer islands (lowest). Without considering the outer islands, there was a 1.36-fold difference in population-adjusted costs of antineoplastic drugs between the southern region and the eastern region and a 1.66-fold difference in population-adjusted costs of targeted therapies between the southern region and the eastern region in 2012.

Results from univariate and multivariate regression models (without considering the outer lands) are shown in Tables 2 and 3. For antineoplastic agents, year and region were significantly associated with their costs but they were not associated with the use of these drugs, after adjusting for covariates. Using the southern region as the reference, there was no significant difference in use of antineoplastic agents between regions, but their costs were significantly lower in other regions. Average age of cancer patients, percentage of male cancer patients, and population density were also significantly and positively associated with costs of antineoplastic drugs. For targeted therapies in particular, year and region were not significantly associated with their use although year was associated with their costs. Using the southern region as the reference, there was no significant difference in use and costs of targeted therapies between regions. Percentage of male cancer patients was significantly and positively associated with use and costs of targeted therapies.

**Table 2:** Results of ANOVA assessing association between region and use and costs of antineoplastic agents in Taiwan

Dependent Variable	Independent Variables and Confounders	Unadjusted F	*P*-value	Adjusted F	*P*-value
Population adjusted prescription volume of antineoplastic agents per 100,000 people					
	Region	100.019	**<** ***0.001***	3.456	0.131
	Year	166.477	**<** ***0.001***	1.672	0.309
	Average Age			0.473	0.529
	% of Male			1.869	0.243
	Population density			0.297	0.615
	Physician density			0.352	0.585
	Cancer incidence			0.003	0.959
Population adjusted cost of antineoplastic agents per 100,000 people					
	Region	61.595	**<** ***0.001***	13.735	***0.014***
	Year	145.303	**<** ***0.001***	132.461	**<** ***0.001***
	Average Age			64.382	***0.001***
	% of Male			93.412	***0.001***
	Population density			37.111	***0.004***
	Physician density			1.332	0.313
	Cancer incidence			4.128	0.112
Population adjusted prescription volume of TT per 100,000 people					
	Region	19.165	**<** ***0.001***	1.295	0.391
	Year	51.340	**<** ***0.001***	6.103	0.057
	Average Age			0.152	0.717
	% of Male			8.368	***0.044***
	Population density			0.546	0.501
	Physician density			0.701	0.449
	Cancer incidence			0.001	0.978
Population adjusted cost of TT per 100,000 people					
	Region	13.449	***0.001***	2.557	0.193
	Year	52.473	**<** ***0.001***	16.615	***0.010***
	Average Age			0.263	0.635
	% of Male			18.796	***0.012***
	Population density			0.615	0.477
	Physician density			1.471	0.292
	Cancer incidence			0.640	0.469

*TT* targeted therapies; Average Age = average age of cancer patients; % of Male = percentage of male cancer patients; Population density = number of population divided by geographic area; Physician density = number of physician divided by number of population in that region per 100,000 people; bold and italic = significant

**Table 3:** Results of multivariate regression model assessing association between region and use and costs of antineoplastic agents in Taiwan

Dependent Variable	Independent Variables and Confounders	Unadjusted t	*P*-value	Adjusted t	*P*-value
Population adjusted prescription volume of antineoplastic agents per 100,000 people					
	Southern region	reference		reference	
	Northern region	-1.146	0.281	-0.565	0.602
	Midwestern region	-10.056	**<** ***0.001***	-0.733	0.504
	Eastern region	-14.653	**<** ***0.001***	0.409	0.703
	Year 2012	reference		reference	
	Year 2011	-5.676	**<** ***0.001***	-1.392	0.236
	Year 2010	-12.803	**<** ***0.001***	-1.264	0.275
	Year 2009	-21.100	**<** ***0.001***	-1.554	0.195
	Average Age			-0.688	0.529
	% of Male			1.367	0.243
	Population density			0.545	0.615
	Physician density			0.593	0.585
	Cancer incidence			-0.055	0.959
Population adjusted cost of antineoplastic agents per 100,000 people					
	Southern region	reference		reference	
	Northern region	-0.703	0.500	-5.797	***0.004***
	Midwestern region	-4.448	***0.002***	- 4.963	***0.008***
	Eastern region	-12.106	***<0.001***	- 5.519	***0.005***
	Year 2012	reference		reference	
	Year 2011	-4.831	***0.001***	-8.271	***0.001***
	Year 2010	-10.996	***<0.001***	-7.521	***0.002***
	Year 2009	-19.753	***<0.001***	-10.691	***<0.001***
	Average Age			8.024	***0.001***
	% of Male			9.665	***0.001***
	Population density			6.092	***0.004***
	Physician density			-1.154	0.313
	Cancer incidence			2.032	0.112
Population adjusted prescription volume of TT per 100,000 people					
	Southern region	reference		reference	
	Northern region	-0.811	0.438	-0.726	0.508
	Midwestern region	-3.498	***0.007***	-0.107	0.920
	Eastern region	-6.858	***<0.001***	0.567	0.601
	Year 2012	reference		reference	
	Year 2011	-5.674	***<0.001***	-2.343	0.079
	Year 2010	-9.248	***<0.001***	-1.388	0.238
	Year 2009	-11.659	***<0.001***	-1.247	0.281
	Average Age			-0.389	0.717
	% of Male			2.893	***0.044***
	Population density			0.739	0.501
	Physician density			0.838	0.449
	Cancer incidence			-0.030	0.978
Population adjusted Cost of TT per 100,000 people					
	Southern region	reference		reference	
	Northern region	-0.467	0.652	-0.760	0.490
	Midwestern region	-2.957	***0.016***	-0.338	0.752
	Eastern region	-5.631	***<0.001***	0.489	0.650
	Year 2012	reference		reference	
	Year 2011	-5.320	***<0.001***	-3.813	***0.019***
	Year 2010	-8.603	***<0.001***	-2.238	0.089
	Year 2009	-12.036	***<0.001***	-2.323	0.081
	Average Age			0.512	0.635
	% of Male			4.335	***0.012***
	Population density			0.784	0.477
	Physician density			1.213	0.292
	Cancer incidence			-0.800	0.469

*TT* targeted therapies; Average Age = average age of cancer patients; % of Male = percentage of male cancer patients; Population density = number of population divided by geographic area; Physician density = number of physician divided by number of population in that region per 100,000 people; bold and italic = significant

### Market share of targeted therapies

Apart from the outer islands, the market share of targeted therapies by prescription volume for northern, midwestern, southern, and eastern regions of Taiwan was around 5% (4.42%~6.70%) in 2012, but they accounted for over 25% of costs for all antineoplastic agents (Table 4). The yearly market share of targeted therapies by prescription volume for each region increased almost 2-fold (1.84-1.90) between 2009 and 2012 without considering the outer islands. The southern region had the highest market share by prescription volume (6.70%) in 2012, followed by the northern (6.39%), midwestern (5.94%), and eastern regions (4.42%), and the outer islands (0.57%). This represents about 11.70-fold difference between regions if the outer islands were included; without considering the outer islands, there was only a 1.52-fold difference between regions.

**Table 4:** Yearly market share by prescription volume and costs of antineoplastic agents by region (2009-2012)

Region	Year	Market share by prescription volume and Costs of targeted therapies						
		Population	Number of prescription for TT	Number of prescription for all antineoplastic agents	Market share by prescription volume (%)	Cost of TT (US$)	Cost of all antineoplastic agents (US$)	Market share by cost (%)
Northern	2009	10,794,022	41,563	1,200,447	3.46	44,727,733	245,327,406	18.23
	2010	10,852,146	53,085	1,352,126	3.93	64,066,867	298,340,256	21.47
	2011	10,919,415	74,409	1,539,869	4.83	80,098,867	335,228,190	23.89
	2012	10,991,480	107,447	1,681,041	6.39	108,328,729	369,543,105	29.31
Midwestern	2009	5,201,847	16,182	517,142	3.13	17,550,735	105,008,551	16.71
	2010	5,199,849	22,116	587,463	3.76	25,919,867	130,844,276	19.81
	2011	5,203,796	28,424	641,058	4.43	31,768,038	148,131,812	21.45
	2012	5,215,948	41,136	691,949	5.94	45,052,173	168,569,414	26.73
Southern	2009	6,446,720	26,808	735,122	3.65	27,431,583	143,013,766	19.18
	2010	6,433,342	33,836	834,331	4.06	37,988,591	178,074,087	21.33
	2011	6,422,584	45,262	904,722	5.00	48,387,511	201,906,589	23.97
	2012	6,422,531	65,905	983,272	6.70	66,284,539	223,610,507	29.64
Eastern	2009	573,461	1,255	52,193	2.40	1,872,532	9,729,754	19.25
	2010	569,478	1,736	60,197	2.88	2,248,464	12,178,410	18.46
	2011	565,128	2,415	66,179	3.65	2,951,519	14,101,363	20.93
	2012	561,442	3,047	68,875	4.42	3,493,182	14,396,745	24.26
Outer Islands	2009	103,722	1	2,431	0.04	53	170,837	0.03
	2010	107,308	0	3,261	0.00	0	316,458	0.00
	2011	113,989	1	3,654	0.03	649	387,676	0.17
	2012	124,421	26	4,539	0.57	17,180	481,335	3.57

*TT* targeted therapies; Market share by prescription volume of targeted therapies (%) = number of prescription of targeted therapies for the region/number of prescription of all antineoplastic agents for the same region; Market share by costs of targeted therapies (%) = cost of targeted therapies for the region/cost of all antineoplastic agents for the same region

The yearly market share by costs of targeted therapies for each region also grew around 1.5-fold (1.26-1.61) from 2009 to 2012. The southern region had the highest market share by costs (29.64%) in 2012, followed by the northern (29.31%), midwestern (26.73%), and eastern regions (24.26%), and the outer islands (3.57%). It represents about 8.30-fold difference between regions if the outer islands were included; without considering the outer islands, there was only a 1.22-fold difference between regions.

### Trends in use of targeted therapies

**Fig. 1:**
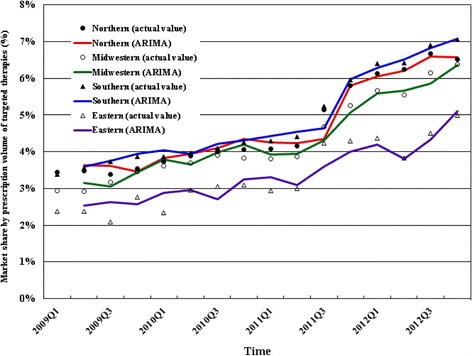
2009-2012 trend in market share of targeted therapies by prescription volume among total antineoplastic agents by region in Taiwan

**Fig. 2:**
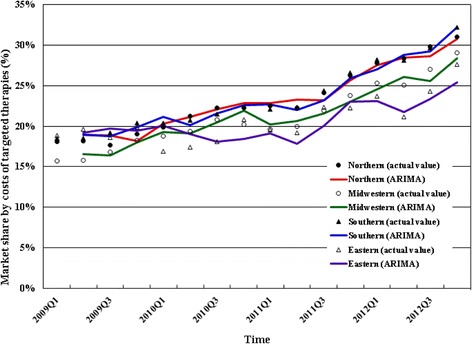
2009-2012 trend in proportion of costs for targeted therapies among total antineoplastic agents by region in Taiwan

We estimated 2009-2012 trends in quarterly market shares by prescription volume and costs for targeted therapies among all antineoplastic agents by region. Figure 1 shows 2009-2012 trends in market share of targeted therapies by prescription volume by region. In the southern region (region with the highest rate in 2012), the quarterly market share of targeted therapies by prescription volume rose rapidly from 3.39% in the first quarter of 2009 to 7.05% in the fourth quarter of 2012. Figure 2 shows 2009-2012 trends in market share by costs by region. In the southern region (region with the highest rate in 2012), the quarterly market share by costs of targeted therapies rose rapidly from 18.51% in the first quarter of 2009 to 32.19% in the fourth quarter of 2012.

## Discussion

Our present study is the first study that estimated the longitudinal trend in use and costs of antineoplastic agents including targeted therapies for cancer treatment by geographic region in Taiwan. Overall our findings do not indicate a significant association between region and use of antineoplastic drugs or use of targeted therapies, after controlling for confounders. Our results suggest an association between region and costs of antineoplastic drugs but we did not find an association between region and costs of targeted therapies. We observed small differences in population-adjusted use of all antineoplastic drugs and a 2-fold difference in use of targeted therapies between regions except the outer islands; the outer islands had substantially lower use of antineoplastic drugs.

Previous studies suggested cancer incidence and physician density as determinants of prescription [42, 43]. However, we did not identify these as significant predictors of use and costs of antineoplastic agents or of targeted therapies, after adjusting for covariates including region. It might be because cancer incidence was similar across regions. Access to oncologists might be more important than physician density in the case of access to cancer treatments but there is a lack of information to estimate oncologist density. Our study suggests that average age of cancer patients and percentage of male cancer patients were associated with costs of antineoplastic drugs but they were not associated with use of antineoplastic drugs. These variables might be related to dosing which would impact the overall treatment costs but not the prescription. Furthermore, percentage of male cancer patients was associated with both use and costs of targeted therapies. This is not surprising because lung cancer and colorectal cancer, two of the top three cancers accounting for targeted therapy use have higher prevalence in men [33, 39].

The difference in use of antineoplastic drugs and targeted therapies between the outer islands and other regions were greater than the difference in cancer incidence between these regions. In addition, there was almost no use of targeted therapies until 2012 in the outer islands, suggesting late adoption of new treatments in this region. It may reflect the limited availability of specialist care and hospitals in the outer islands, and patients with cancer in this region may seek and receive cancer treatments in hospitals in other regions instead [44]. To reduce the gap in access to targeted therapies and other antineoplastic drugs between the outer islands and other regions, more efforts might be needed to improve accessibility to care, including diagnosis and treatment of cancer, in the outer islands, or government support for people to travel to the southern region (the region closest to the outer islands) to receive care.

Our findings show that while the market share of targeted therapies by prescription volume increased from 3% in 2009 to 6% in 2012 without considering the outer islands, their market share by costs increased from about 20% in 2009 to about 30% in 2012. Growing trends in use and costs of targeted therapies for cancer treatment is likely to continue in Taiwan and in all of its regions, which may cause increasing economic burden. In Taiwan, similar to many other countries, health technology assessment to restrict coverage, prior authorization, and higher out-of-pocket costs by patients have been used to relief some economic burden for the national health system. The high cost of targeted therapies may continue to be a barrier to access for patients with cancer [45, 46]. Increasing economic burden on cancer care overall due to growing use of high-cost targeted therapies challenges the sustainability of the universal insurance system because there might be ‘trade-offs’ to be made with reimbursements for non-cancer services and treatments. It is important that policymakers revisit the pricing and reimbursement structures for targeted therapies due to their high prices especially because some therapies have little rigorous effectiveness and safety evidence [47–50]. Strategies are needed to ensure patient access to effective health services and treatments without overspending the healthcare budget.

The high costs of cancer targeted therapies and their increasing use for many cancers are issues of growing attention globally because cancer incidence is expected to rise worldwide, from 14 million newly diagnosed patients annually in 2012 to 22 million within the next two decades [51]. With the high costs of cancer care, research should investigate regional inequities in treatment that can results in inequities in health outcomes. Such research is important to inform policy and program implementation to reduce inequities.

There are some limitations to this study. First, this study aimed to examine the recent trends in utilization of antineoplastic drugs particularly cancer targeted therapies by geographic region in Taiwan. We did not analyze the prescribing patterns of antineoplastic drugs and targeted therapies by patient/physician characteristics, and by other environmental factors (number of hospitals, cancer clinics etc). Second, this study examined the trend in overall targeted therapy use, we did not categorize targeted therapies by their pharmacological classification (e.g., protein kinase inhibitors) and indications (types of cancers). Third, our model included physician density by region as a confounder. It would be more precise to estimate and adjust for oncologist density by region; however, this information was not available. Nonetheless, this study should provide a basis for additional research. Further research is needed on geographic variations in cancer screening and diagnosis in Taiwan, and in use of antineoplastic drugs particularly targeted therapies considering other factors such as patient characteristics, physician preferences, and classes of targeted therapies. Studies are also warranted to better understand access to cancer care and treatment in the outer islands.

## Conclusion

This study estimated the nationwide, longitudinal trend in use and costs of antineoplastic drugs by geographic region in Taiwan with a focus on targeted therapies. Overall, we found some geographic variations in use of antineoplastic drugs in Taiwan; use was notably low in the outer islands. Similar to other countries, growing trend in use and costs of targeted therapies for cancer treatment is likely to continue in Taiwan, which may cause increasing economic burden. Strategies might be needed to ensure access to cancer care in each region as economic burden of cancer care increase due to growing use of high-cost targeted therapies.

## Appendix 1

**Table 5:** Classification of regions in this study

Region	Administrative divisions
Northern	Taipei City, New Taipei City, Keelung City, Hsinchu City, Hsinchu County, Taoyuan City, Yilan County, Miaoli County
Midwestern	Taichung City, Changhua County, Nantou County
Southern	Tainan City, Yunlin County, Chiayi City, Chiayi County, Kaohsiung City, Pingtung County
Eastern	Taitung County, Hualien County
Outer islands	Kinmen County, Penghu County, Lienchiang County

## Appendix 2

**Table 6:** Cancer Incidence, Population Density and Physician Density by year and region

Region	Year	Cancer Incidence (per 100,000 people)	Population Density (people per hectare)	Physician Density (doctor per 100,000 people)
Northern	2009	366	12.38	167.94
	2010	381	12.26	171.49
	2011	384	12.32	176.16
	2012	399	12.40	179.87
Midwestern	2009	372	6.34	163.54
	2010	377	6.31	167.86
	2011	392	6.32	172.75
	2012	403	6.34	175.21
Southern	2009	402	6.64	159.03
	2010	421	6.58	163.83
	2011	431	6.56	167.65
	2012	452	6.55	170.88
Eastern	2009	387	0.72	173.33
	2010	413	0.72	174.90
	2011	425	0.71	179.96
	2012	447	0.71	185.42
Outer Islands	2009	189	6.97	92.53
	2010	197	7.11	97.93
	2011	224	7.34	90.46
	2012	238	7.74	95.40

## Abbreviations

% of Male: Percentage of male cancer patients
ANOVA: Analysis of variance
ARIMA: Autoregressive integrated moving average
ATC: Anatomical Therapeutic Chemical
Average Age: Average age of cancer patients
ICD-9: International Classification of Diseases, 9th edition
NHIRD: National Health Insurance Research Database
TT: Targeted therapies
